# Prediction of fresh and ripened cheese yield using detailed milk composition and udder health indicators from individual Brown Swiss cows

**DOI:** 10.3389/fvets.2022.1012251

**Published:** 2022-10-13

**Authors:** Elena Mariani, Massimo Malacarne, Claudio Cipolat-Gotet, Alessio Cecchinato, Giovanni Bittante, Andrea Summer

**Affiliations:** ^1^Department of Veterinary Science, University of Parma, Parma, Italy; ^2^Department of Agronomy, Food, Natural Resources, Animals and Environment (DAFNAE), University of Padova, Legnaro, Italy

**Keywords:** phenomics, predictive equation, cheese-making, protein fractions, udder health indicators, breeding programs, sustainability

## Abstract

The composition of raw milk is of major importance for dairy products, especially fat, protein, and casein (CN) contents, which are used worldwide in breeding programs for dairy species because of their role in human nutrition and in determining cheese yield (%CY). The aim of the study was to develop formulas based on detailed milk composition to disentangle the role of each milk component on %CY traits. To this end, 1,271 individual milk samples (1.5 L/cow) from Brown Swiss cows were processed according to a laboratory model cheese-making procedure. Fresh %CY (%CY_CURD_), total solids and water retained in the fresh cheese (%CY_SOLIDS_ and %CY_WATER_), and 60-days ripened cheese (%CY_RIPENED_) were the reference traits and were used as response variables. Training-testing linear regression modeling was performed: 80% of observations were randomly assigned to the training set, 20% to the validation set, and the procedure was repeated 10 times. Four groups of predictive equations were identified, in which different combinations of predictors were tested separately to predict %CY traits: (i) basic composition, i.e., fat, protein, and CN, tested individually and in combination; (ii) udder health indicators (UHI), i.e., fat + protein or CN + lactose and/or somatic cell score (SCS); (iii) detailed protein profile, i.e., fat + protein fractions [CN fractions, whey proteins, and nonprotein nitrogen (NPN) compounds]; (iv) detailed protein profile + UHI, i.e., fat + protein fractions + NPN compounds and/or UHI. Aside from the positive effect of fat, protein, and total casein on %CY, our results allowed us to disentangle the role of each casein fraction and whey protein, confirming the central role of β-CN and κ-CN, but also showing α-lactalbumin (α-LA) to have a favorable effect, and β-lactoglobulin (β-LG) a negative effect. Replacing protein or casein with individual milk protein and NPN fractions in the statistical models appreciably increased the validation accuracy of the equations. The cheese industry would benefit from an improvement, through genetic selection, of traits related to cheese yield and this study offers new insights into the quantification of the influence of milk components in composite selection indices with the aim of directly enhancing cheese production.

## Introduction

As cheese consumption per capita continues to rise in Europe, North America, and Oceania (1), the dairy sector is looking for new sustainable ways to improve the cheese-making ability of milk. Cheese yield is usually expressed as a percentage (%CY) representing the amount of cheese produced from 100 kg of milk (2). This trait is fundamental not only to determine the profitability of dairy industries and farmers, but also to monitor the sustainability of the dairy chain. The variability in %CY is affected by many factors relating to milk quality and processing characteristics. The composition of the raw milk is of major importance, especially the content of fat and protein (or caseins) (3), which are used worldwide in breeding programs for dairy species (4) not only because of their nutritional role, but also because of their effect on %CY (5). The %CY is traditionally determined from bulk milk at the dairy industry level (6–8), but for research and genetic purposes, it can also be determined at the individual level through laboratory model cheese-making procedures (9, 10). These techniques provide the opportunity to study individual animal variability using small quantities of milk (e.g., from 1.7 to 7,000 mL) (11, 12) in procedures consisting of a series of highly controlled, standardized steps (e.g., cutting time, heating temperature). They also offer the possibility to measure the recovery of nutrients (%REC) in the curd throughout the weight and the composition of processed milk and whey. Few studies have estimated the heritability of measured cheese-making traits (5), including other dairy species (13, 14). This is mainly because of high costs and limitations due to the labor intensity of each step in the cheese-making procedure at the individual animal level (10). To overcome the economic and practical obstacles of individual analysis, Fourier-transform infrared spectroscopy has been used to investigate the suitability of predicted %CY and %REC traits at the population level (15, 16). However, unlike the predictions of milk chemical components (i.e., milk fatty acids and protein fractions) (17, 18), predictions of the technological features of milk, such as cheese-making traits, are often not sufficiently accurate to be classified as gold standard (19), so their application at the population level is still under review.

These limitations can be overcome using predictive formulas developed on the basis of the contribution of specific milk components to %CY. Since the early 1900s, many predictive %CY formulas have been constructed based on milk fat and protein content. These formulas are useful for (i) predicting %CY of specific cheeses, for example Cheddar (20) and Parmigiano Reggiano (21), and (ii) comparing predicted and measured %CY in order to monitor the efficiency of the cheese-making process (i.e., precision of weights and measurements) (22). As previously mentioned, milk fat and protein are currently used worldwide in the selection programs of dairy cattle, and in some countries their economic weights are based on their contribution to cheese yield. However, other milk components are also recognized as influencing %CY and %REC traits, such as somatic cell count (SCC) and lactose, which are used as indicators of mastitis (23). Nevertheless, it is well known that the levels of efficiency by which milk nutrients are transferred to the curd and the cheese vary according to the breed of the cow (24), and hence can be only partially explained by differences in coagulation, curd firming and syneresis (25). In fact, the differences can be explained mainly in terms of the different milk protein profiles (26). Brown Swiss milk is usually considered particularly suitable for cheese production due to its fat and protein composition (27).

If each milk protein fraction has different effects on cheese-making efficiency as previously found by Cipolat-Gotet et al. (28), we speculate that %CY prediction formulas based on protein or total casein (CN) will not be able to completely explain these effects. It is therefore essential to understand the role of the detailed milk composition, including milk protein fractions and udder health indicators, in the retention of milk nutrients and water in the curd and cheese, and to quantify the relative importance of these nutrients. This information could also be used to refine the selection goals for a dairy population, and to establish more precisely the economic weights of milk components in selection indices. For these reasons, the general aim of the present study was to identify and quantify the effects of detailed milk components on %CY. The specific objectives were: (i) to study %CY in terms of fresh cheese (%CY_CURD_), milk solids (%CY_SOLIDS_) and water (%CY_WATER_) retained in the curd, and ripened cheese (%CY_RIPENED_); (ii) to quantify the effects on %CY of the milk components mainly retained in cheese (fat, protein and/or CN); (iii) to quantify the effects on %CY of milk traits mainly related to udder health [lactose and somatic cell score (SCS)]; and (iv) to quantify the effects on %CY of single milk protein fractions and their relative importance [CN fractions, whey proteins, and nonprotein nitrogen (NPN)].

## Materials and methods

### Ethical statement

All the dairy cows involved in this study were reared in commercial private farms and were not subjected to any invasive procedures. Milk samples from dairy cows used for the project were collected by technicians of breeders associations during routine milking within current milk-recording schemes (ICAR, International Committee for Animal Recording) and hence certified by local authorities.

### Experimental design, animals and milk sampling

The present study is part of the Cowability-Cowplus projects. The milk from 1,271 Brown Swiss cows was collected once during the evening milking and divided in 3 subsamples per each cow. The whole sampling collection took place over the course of one year. The sampled cows represented different stages of lactation (25–388 days in milk) and parities (1–5) and were clinically healthy. Cows belonged to 85 herds (15 cows per herd, with a few exceptions) selected from 610 farms located in Trento Province (Italy) and representing different environments and dairy farming systems as described by Berton et al. (29). Briefly, the dairy farming systems were classified into 4 categories, 1 traditional and 3 modern types, which differed for the use and type of total mixed ration.

Among the 3 subsamples collected for each animal, one was analyzed for composition at the Milk Quality Laboratory of the Trento Breeders Association (Trento, Italy), and the others were transported to the Milk Laboratory of DAFNAE (Department of Agronomy, Food, Natural Resources, Animals and Environment) of the University of Padova (Legnaro, Padua, Italy) for cheese-making and quantification of the milk protein fractions.

### Milk analyses and processing

#### Milk gross composition

Individual raw full-fat milk samples (50 mL) were analyzed within 20 h from milking for gross composition (protein, casein, fat, lactose, and total solids) with a MilkoScan FT6000 (Foss, Hillerød, Denmark) calibrated according to the reference methods described by Cipolat-Gotet et al. (1). SCC values were obtained with a Fossomatic FC counter (Foss, Hillerød, Denmark) then converted into SCS using the formula SCS = log_2_(SCC/100,000) + 3 (30).

#### Milk protein fractions

Individual milk samples (2 aliquots of 1 mL each per cow) were mixed with preservative (bronopol, 2-bromo-2-nitropropan-1,3-diol, 0.6:100 vol/vol) to prevent microbial development, frozen at −20°C in portable chilling devices immediately after collection, then stored at −80°C until analysis. Frozen individual milk aliquots were prepared following the method proposed by Bobe et al. (31). The contents of the CN fractions (α_S1_-, α_S2_-, β-, and κ-CN) and whey proteins (β-LG and α-LA) were assessed by the validated reversed-phase HPLC method (32). The remaining NPN content was estimated as the difference from the total milk nitrogen content.

#### Model cheese-making and cheese yield traits

Individual milk samples were processed within 20 h from milking according to the model cheese-making method described in detail by Cipolat-Gotet et al. (33). Briefly, 1,500 mL of milk from each cow were heated to 35°C in a stainless-steel micro-vat, thermophilic starter culture was added, milk was mixed with rennet and monitored for gelation time. The starter was an industrial freeze-dried formulation of thermophilic lactic bacteria (Delvo-Tec TS-10A DSL; DSM Food Specialties, Delft, The Netherlands). At a fixed time after gelation (10 min) each curd was cut with a vertical crosscut centered on the vertical axis of the vat. Five min after the first cut, the curd was reduced to cubes of about 1 cm^3^. After a further 5 min, the curd was separated from the whey and suspended on a cheese mold for 30 min over the whey-containing vat and turned every 2 min to facilitate draining. The curd was then pressed for 60 min at 250 kPa, turning every 20 min, and salted for 60 min in liquid brine at a saturation of 20% NaCl. The whey collected from each vat was also weighed, sampled, and analyzed for fat, protein, lactose, and total solids content with a MilkoScan FT2 (Foss, Hillerød, Denmark). At the end of the cheese-making process and after brining, each cheese wheel was weighed. Curd components (fat, protein, and total solids) were measured as the difference in composition between the milk before processing and the whey. All the cheeses were then ripened at 15°C and 85% relative humidity for the first month, then at 12°C and the same relative humidity for the second month (a total of 60 days).

With the aforementioned measurements we were able to obtain four %CY traits. The classical formulas for %CY at 0 d (fresh curd), and at 60 d after ripening were calculated as follows:


$$\begin{array}{l} \%CY_{CURD=}\frac{weight\;of\;wheel\;at\;0\;d\;(g)}{weight\;of\;milk\;(g)}\times 100\;\\ \%CY_{RIPENED=}\frac{weight\;of\;wheel\;at\;60\;d\;(g)}{weight\;of\;milk\;(g)}\times 100\; \end{array}$$

Cheese yield was also calculated in terms of total solids (TS) and water retained in the fresh curd, as follows:


$$\begin{array}{l} \%CY_{SOLIDS=}\frac{milk\;TS\;(g)\text{–}whey\;TS\;(g)}{weight\;of\;milk\;(g)}\times 100\;\\ \%CY_{WATER=}\frac{milk\;water\;(g)\text{–}whey\;water\;(g)}{weight\;of\;milk\;(g)}\times 100\; \end{array}$$

Where milk and whey water were obtained as differences with respective TS.

### Statistical analysis

#### Editing

Before the statistical analysis, all trait values (milk composition, protein fractions, and %CY traits) falling outside 3 standard deviations (SD) of the mean were removed to exclude outliers, so that the results shown in Table 1 are already presented without outliers.

**Table 1:** Descriptive statistics of daily milk yield, milk components (gross composition, somatic cell score and protein fractions) and cheese yield.

**Trait^a^**	***N***	**Mean**	**CV^b^, %**	**p5^c^**	**p95^c^**
dMY, kg/d	1,250	24.40	32	12.30	37.90
Milk Composition					
Fat, %	1,229	4.22	21	3.14	5.42
Protein, %	1,229	3.71	11	3.03	4.43
Casein, %	1,229	2.89	11	2.38	3.44
αS1-CN, %	1,233	0.95	15	0.75	1.18
αS2-CN, %	1,233	0.34	18	0.25	0.44
β-CN, %	1,233	1.19	14	0.95	1.44
κ-CN, %	1,233	0.35	21	0.22	0.46
α-LA, %	1,232	0.09	22	0.06	0.12
β-LG, %	1,232	0.32	23	0.21	0.45
NPN, %^2^	1,231	0.41	25	0.24	0.58
Lactose, %	1,229	4.85	5	4.50	5.13
TS, %	1,271	13.90	8	12.40	15.60
SCS, unit	1,229	2.98	62	0.21	6.20
Cheese Yield, %					
%CY_CURD_	1,257	15.00	13	12.00	18.30
%CY_SOLIDS_	1,247	7.22	13	5.77	8.82
%CY_WATER_	1,251	7.80	16	5.85	9.95
%CY_RIPENED_	1,224	8.73	13	6.99	10.61

a dMY, daily milk yield, kilogram per day; NPN, nonprotein nitrogen; TS, total solids; SCS, somatic cell score.

b CV, coefficient of variation.

c p5, 5th percentile; p95, 95th percentile.

#### Regression models

Linear regression models were tested separately for predicting %CY traits (%CY_CURD_, %CY_SOLIDS_, %CY_WATER_ and %CY_RIPENED_) using different combinations of milk components as predictors selected on the basis of their correlations, technological roles and effects on cheese production (18, 34, 35). To quantify the weight of each nutrient on %CY the regressions tested included the major milk nutrients transferred to cheese. Four groups of predictive equations were identified, in which different combinations of predictors were tested separately to predict %CY traits:

(i) basic composition, i.e., fat, protein and casein, tested individually and combined;
(ii) udder health indicators (UHI), i.e., fat + protein or casein + lactose and/or SCS;
(iii) detailed protein profile, i.e., fat + protein fractions, precisely
  a. casein fractions
  b. casein fractions + whey proteins
  c. casein fractions + whey proteins + NPN compounds
(iv) detailed protein profile + UHI, i.e., fat + protein fractions + NPN compounds and/or lactose and/or SCS.

For all the %CY traits, we tested regression models both with and without intercept, although the results from the models with intercept are not reported as our main goal was to quantify the real contribution of each of the predictors to %CY. However, the fitting statistics between the models with and without intercept were comparable (data not shown). Values of the adjusted coefficients of determination of calibration (${\text{adjR}}_{\text{CAL}}^{2}$) were calculated using the following formula:


$$adjR^{2}{}_{CAL=}1-\frac{\left(1-R^{2})(N-1\right)}{N-p-1}$$

Where R^2^ is the sample R-squared, N is the total sample size and p is the number of independent variable.

For all the predictors, *P*-values were not reported since they were always lower than 0.001. Multicollinearity for two groups of variables was checked by evaluation of tolerance, variance inflation factor, Eigen values and condition index (Supplementary Table 1). The two groups included the following predictor variables: group (1) fat, protein, lactose and SCS; group (2) fat, protein fractions, NPN compounds, lactose and SCS. The results obtained from those tests evidenced the absence of multicollinearity among predictors of each group (Supplementary Table 1).

#### Validation

The accuracies of the %CY predictive formulas were assessed through a training-testing procedure. A training data set (80% of the total observations) was used to build the predictive equations, and a testing data set (20% of the total) was used as validation. Observations were randomly assigned to the training and testing sets, and the training-testing procedure was repeated 10 times for each of the %CY traits, changing the training and testing set samples each time. For each of the 10 training-testing tests of the prediction procedure of a given trait, the observed and the predicted values of the testing data set were used to calculate the coefficient of determination of validation (${\text{R}}_{\text{VAL}}^{2}$) and the root mean square error of validation (RMSE_VAL_). The beta coefficient of each predictor, ${\text{R}}_{\text{VAL}}^{2}$ and RMSE_VAL_ for each trait are presented as the average of the 10 training-testing replicates carried out.

The ${\text{adjR}}_{\text{CAL}}^{2}$ values were similar to the ${\text{R}}_{\text{VAL}}^{2}$ values highlighting the absence of over-fitting and multicollinearity of the proposed regression models. The ${\text{adjR}}_{\text{CAL}}^{2}$ values were shown in Tables 2–4. Attention was given to ${\text{R}}_{\text{VAL}}^{2}$ as it provides information on the effectiveness of prediction when applied externally (i.e., population level) and, therefore, considered more important for the purpose of this study.

**Table 2:** Regression coefficients and fitting statistics (${\text{adjR}}_{\text{CAL}}^{2}$, ${\text{R}}_{\text{VAL}}^{2}$ and RMSE_VAL_) of predicting fresh curd (%CY_CURD_), cheese solids (%CY_SOLIDS_), water retained in the curd (%CY_WATER_) and ripened cheese (%CY_RIPENED_) of models based on fat, protein and total casein content of processed milk.

	**Models with a single nutrient:**			**Models with combination of nutrients:**	
	**Fat**	**Protein**	**Casein**	**Fat** **+ Protein**	**Fat** **+ Casein**
**%CY**_**CURD**_					
*Regression coefficients*					
Fat, %	3.50	–	–	0.97	0.88
Protein, %	–	4.04	–	2.95	–
Casein, %	–	–	5.19	–	3.91
*Fitting statistics*					
${\text{adjR}}_{\text{CAL}}^{2}$	0.27	0.48	0.52	0.59	0.61
${\text{R}}_{\text{VAL}}^{2}$	0.29	0.49	0.53	0.60	0.62
RMSE_VAL_, %	2.21	1.41	1.34	1.22	1.18
**%CY**_**SOLIDS**_					
*Regression coefficients*					
Fat, %	1.70	–	–	0.82	0.80
Protein, %	–	1.94	–	1.02	–
Casein, %	–	–	2.49	–	1.33
*Fitting statistics*					
${\text{adjR}}_{\text{CAL}}^{2}$	0.56	0.42	0.43	0.75	0.75
${\text{R}}_{\text{VAL}}^{2}$	0.57	0.42	0.43	0.75	0.75
RMSE_VAL_, %	0.81	0.75	0.73	0.47	0.47
**%CY**_**WATER**_					
*Regression coefficients*					
Fat, %	1.81	–	–	0.19	0.13
Protein, %	–	2.10	–	1.88	–
Casein, %	–	–	2.69	–	2.51
*Fitting statistics*					
${\text{adjR}}_{\text{CAL}}^{2}$	0.05	0.28	0.31	0.29	0.31
${\text{R}}_{\text{VAL}}^{2}$	0.06	0.31	0.33	0.31	0.33
RMSE_VAL_, %	1.63	1.07	1.05	1.07	1.04
**%CY**_**RIPENED**_					
*Regression coefficients*					
Fat, %	2.04	–	–	0.72	0.66
Protein, %	–	2.35	–	1.54	–
Casein, %	–	–	3.01	–	2.05
*Fitting statistics*					
${\text{adjR}}_{\text{CAL}}^{2}$	0.39	0.51	0.55	0.69	0.72
${\text{R}}_{\text{VAL}}^{2}$	0.38	0.52	0.56	0.69	0.71
RMSE_VAL_, %	1.21	0.79	0.75	0.62	0.60

## Results and discussion

### Descriptive statistics

Variability in milk composition is well known to be a major factor in determining the efficiency of the cheese-making process. Table 1 summarizes the descriptive statistics for single test-day milk yield (dMY), milk components, and %CY traits. The average fat, protein, and lactose contents were 4.22, 3.71, and 4.85%, respectively, with fat having the highest coefficient of variation (CV, 21%). Regarding protein fractions, the casein index, defined as the percentage of casein on total protein, was 77.9%. As expected, β-CN was the predominant casein fraction (41.2%), followed by α_S1_-CN (32.9%), κ-CN (12.1%), and α_S2_-CN (11.8%). Milk NPN was in a ratio of ~11.0% with protein. This group consists mainly of milk urea together with free amino acids and peptides (36). Our results show that the average %CY_CURD_ was 15.0%, %CY_SOLIDS_ was 7.22%, and %CY_WATER_ 7.80%. The contribution of water to the total fresh %CY was therefore around 52.0%, and solids 48.0%. After ripening, the %CY decreased to 8.73% (%CY_RIPENED_).

### Prediction of cheese yield based on fat, protein or casein alone

As it is well known, the addition of rennet triggers the coagulation process and causes the casein micelles to aggregate and form a network which traps the majority of the fat globules. Most of the milk water and soluble compounds (lactose, whey proteins, many minerals, vitamins, etc.) are then expelled during syneresis and constitute the whey. The most important determinants of %CY, therefore, are the casein micelles and fat globules, although factors affecting whey expulsion are also important.

Even though milk fat and protein contents are correlated (in the present study *r* = 32.0%, Supplementary Table 2), if they are not standardized in the milk before cheese-making, their ratio is far from constant, especially in milk from different farming systems, parity, or lactation stages. It would therefore be expected that trying to predict %CY traits using only one milk component would result in merely moderate accuracies.

Using fat as the only predictor of %CY traits, we were able to predict %CY_CURD_ with a regression coefficient of 3.50 (Table 2). This value is expected because the intercept of the prediction equation was fixed at 0.00 and the ratio between %CY_CURD_ (15.00%) and milk fat content (4.22%) was 3.55. Nevertheless, the validation accuracy of this prediction equation was very modest (${\text{R}}_{\text{VAL}}^{2}$ = 0.29) and the corresponding RMSE_VAL_ was high (2.21%). As the ratio between moisture and total solids in this type of fresh model cheese (Table 1) is slightly in favor of the former, the higher regression coefficient of fat (Table 2) for predicting %CY_WATER_ (1.81) than for predicting %CY_SOLIDS_ (1.70), and their sum almost coinciding with the regression coefficient for %CY_CURD_ (3.50) were also expected. Given that lipids, quantitatively, account for a major part of cheese solids in full-fat cheeses, it is not surprising that the validation accuracy of the fat-based equation predicting %CY_SOLIDS_ (${\text{R}}_{\text{ VAL}}^{2}$ = 0.57) was about twice the ${\text{R}}_{\text{ VAL}}^{2}$ previously seen for %CY_CURD_ (${\text{R}}_{\text{VAL}}^{2}$ = 0.29), whereas for %CY_WATER_ it was almost negligible (${\text{R}}_{\text{ VAL}}^{2}$ = 0.06).

Similarly, in the case of the %CY_CURD_ predictive equations based on milk protein or on milk casein, the regression coefficients were also equal to the ratio between the average of %CY_CURD_ and the average of the predicting nutrient (Tables 1 and 2). Moreover, the regression coefficients of %CY_WATER_ were slightly higher than those of %CY_SOLIDS_ (Table 2). The determination coefficient of the protein-based %CY_CURD_ equation (${\text{R}}_{\text{ VAL}}^{2}$ = 0.49) was, instead, much larger than that of the fat-based equation (${\text{R}}_{\text{ VAL}}^{2}$ = 0.29), and even larger in the case of milk casein content as the predictor (${\text{R}}_{\text{VAL}}^{2}$ = 0.53). The slightly lower content of protein in cheese compared with fat explains the lower determination coefficients of the %CY_SOLIDS_ equations based on protein (0.42) and on casein (0.43), compared to when only fat was included as the predictor (0.57). In contrast, the hydrophilic properties of most proteins explain their higher accuracies compared with fat in predicting %CY_WATER_ (0.31 for protein, 0.33 for casein and 0.06 for fat; Table 2).

After ripening, the ratio between moisture and total solids was less variable than in fresh cheese, and more related to chemical composition of cheese, as long as the cheese-making procedure and ripening conditions were constant. This explains why predicting %CY_RIPENED_ always has a greater validation accuracy than predicting %CY_CURD_ (Table 2).

**Figure 1:**
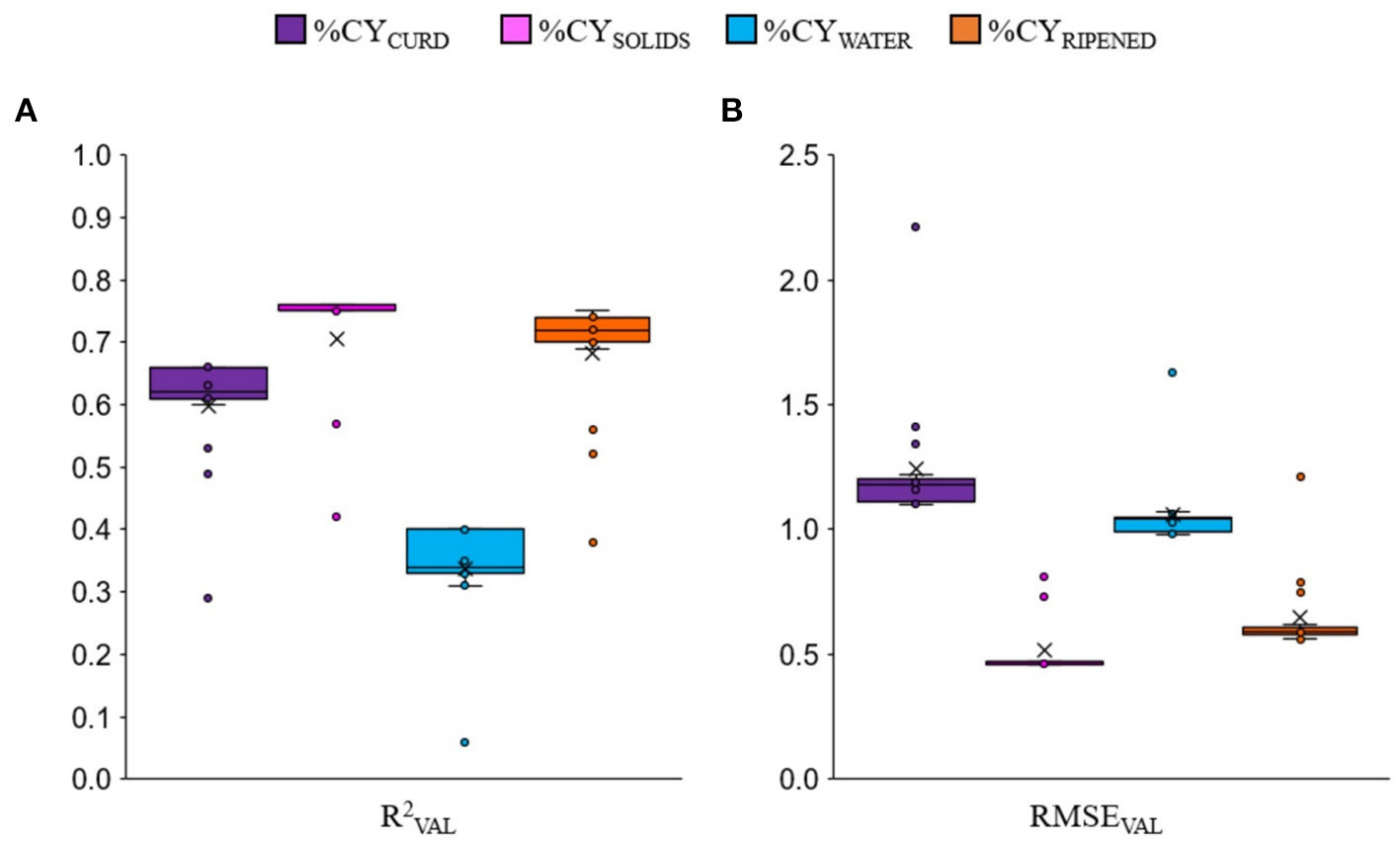
Box-plot of validation accuracy (${\text{R}}_{\text{VAL}}^{2}$) **(A)** and root mean square error (RMSE_VAL_) **(B)** of all the cheese yield prediction equations for fresh curd (%CY_CURD_), cheese solids (%CY_SOLIDS_), water retained in the curd (%CY_WATER_) and 60-days ripened cheese (%CY_RIPENED_) among all the tested linear regression models. The outlier values (circles) are relative only to single-nutrient equations.

The cross-validation approach used in this study showed that, as expected, the prediction equations of the combined-nutrients models that always included milk fat content and protein or CN or protein fractions were on average more accurate than the single-nutrient (fat, protein or CN) models. The box-plots of all the regression models together (Figure 1) clearly show that, on average, predicting %CY_SOLIDS_ was more accurate than predicting %CY_RIPENED_ and %CY_CURD_, and much more accurate than predicting %CY_WATER_. But it is worth noting that, for each %CY trait, the worst validation accuracies (circles = outlier values) were those of the single-nutrient equations, i.e., when fat, protein and casein were tested individually in the formula.

### Prediction of cheese yield based on fat and protein, or casein

Protein and fat are widely used in dairy cattle selection programs and in the milk quality payment system because of their nutritional value and their acknowledged contribution to %CY and the production of other dairy products (i.e., yogurt, butter).

The ${\text{R}}_{\text{VAL}}^{2}$ for the %CY_CURD/SOLIDS/RIPENED_ traits increased and the RMSE_VAL_ decreased when fat was associated with protein or CN (Table 2). This outcome was partly expected, as CN (and indirectly protein) is the main actor in the coagulation process and whey expulsion.

Although fat exerts a large effect on %CY and %REC traits in the processing of milk from many dairy species, such as cattle (37), sheep (38) and goats (39), the regression coefficients of %CY_CURD_ and %CY_SOLIDS_ prediction reflect its own recovery. Indeed, the regression coefficients of fat when combined with protein or CN for predicting %CY_SOLIDS_ were 0.82 and 0.80, respectively (Table 2). These values are slightly lower than the average recovery of milk fat (REC_FAT_ = 89.79%) in the individual model cheese-making procedures carried out in this project (5). As explained in the previous study, the difference could be due to the fact that, as it was not possible to sample the wheels of curd at the beginning of ripening, REC_FAT_ was obtained by difference [(fat in milk – fat in whey)/fat in milk], so the nutrients retained could be slightly overestimated due to small losses in the whey during the procedure and particularly during pressing.

The regression coefficients of fat on the %CY_CURD_ equations were slightly higher (0.97 with protein, 0.88 with CN), consistent with the small regression coefficients obtained for %CY_WATER_ (0.19 and 0.13). It is acknowledged that fat globules contribute to %CY by retaining moisture and acting as a physical obstacle to water (40), but not by exerting any active role in the coagulation process. This means that a small proportion of curd moisture is related to fat content, probably due to the amphiphilic properties of phospholipids and saponified lipids (41).

The regression coefficient of protein for predicting %CY_SOLIDS_ (1.02) was much greater than the recovery of protein during model cheese-making (average REC_PROTEIN_ 78.08%) (5). This is due to the fact that the majority of other solids retained in the curd, especially hydrophilic solids (lactose, minerals, etc.), are proportional to the quantity of whey retained, which in turn is much more proportional to protein (i.e., whey proteins) than to fat (6). For the same reasons, the regression coefficient of casein (1.33) was much larger than the theoretical recovery of casein alone (1.00).

The regression coefficients of protein (2.95) and casein (3.91) when combined with fat for predicting %CY_CURD_ (Table 2) were much higher than unity because of the effect of proteins on the retention of whey in the curd. In fact, the regression coefficients of protein and casein for predicting %CY_WATER_ were 1.88 and 2.51, respectively. In the case of both %CY_CURD_ and %CY_WATER_, the regression coefficient of protein was about 75% that of casein, reflecting their ratio (casein number) in the milk (Table 1).

As previously mentioned, protein or casein alone were better than fat in the prediction of %CY_WATER_, as these components remain bound to water, so that the addition of fat in the prediction equations of %CY_WATER_ did not modify the validation accuracy (Table 2).

Similarly to %CY_SOLIDS_, all the coefficients for %CY_RIPENED_ were lower than those for %CY_CURD_. This can be explained by (i) the reduction in %CY_RIPENED_ (~40%) due to water loss by evaporation from the crust and migration from the inner part of the wheel toward the surface (42); (ii) the microbiological, physical and biochemical reactions occurring during ripening (42, 43), which may act as background noise in the prediction of %CY_RIPENED_. However, the lower regression coefficient of protein and casein was not as pronounced as for %CY_SOLIDS_ due to the water retained in the cheese wheels at the end of the ripening period.

### Contribution of udder health indicators to cheese yield

The inclusion in the statistical model of traits associated with udder health (SCS and lactose) only slightly increased the validation accuracy of the %CY prediction equations (Table 3), although the regression coefficients obtained are useful for increasing our knowledge of the relationships between these traits and the efficiency of the cheese-making process. Lactose percentage and SCC are associated with the udder health status of dairy cows (44–46). During mastitis, the mammary gland tissues are damaged, secretory cell activity is reduced causing a reduction in the synthesis of lactose (47), and in addition the permeability of the membrane increases causing leakage of lactose into the blood stream (23). Milk SCC encompasses a mixture of epithelial cells and leukocytes and has been widely used as an indicator of intramammary infections. SCC is of further importance as a widely accepted parameter for establishing the hygienic quality of raw milk and is currently used in the milk payment system (48, 49). UHI could also have consequences for milk technological properties. High SCC is correlated with reduced fat and lactose contents, but also with an increased level of whey proteins and lower concentrations of caseins (50). Moreover, a variation in the lactose percentage affects the pH of milk and is associated with lower clotting ability (51). All these modifications to the milk composition could cause a reduction in %CY (52), with consequent decrease of the efficiency and sustainability of the whole process.

**Table 3:** Regression coefficients and fitting statistics (${\text{adjR}}_{\text{CAL}}^{2}$, ${\text{R}}_{\text{VAL}}^{2}$ and RMSE_VAL_) of predicting fresh curd (%CY_CURD_), cheese solids (%CY_SOLIDS_) and water retained in the curd (%CY_WATER_), and ripened cheese (%CY_RIPENED_), of models based on fat and protein or casein and on lactose and/or somatic cell score (SCS) of processed milk.

	**Models with lactose:**		**Models with SCS:**		**Models with lactose and SCS:**	
	**Fat** **+protein** **+lactose**	**Fat** **+casein** **+lactose**	**Fat** **+protein** **+SCS**	**Fat** **+casein** **+SCS**	**Fat** **+protein** **+ lactose +SCS**	**Fat** **+casein** **+lactose** **+ SCS**
**%CY**_**CURD**_						
*Regression coefficients*						
Fat, %	0.85	0.81	0.96	0.89	0.86	0.82
Protein, %	2.48	–	3.06	–	2.63	–
Casein, %	–	3.43	–	4.00	–	3.56
Lactose, %	0.47	0.36	–	–	0.41	0.31
SCS, unit	–	–	– 0.13	– 0.09	– 0.10	– 0.08
*Fitting statistics*						
${\text{adjR}}_{\text{CAL}}^{2}$	0.61	0.62	0.60	0.61	0.61	0.62
${\text{R}}_{\text{VAL}}^{2}$	0.62	0.63	0.61	0.62	0.62	0.63
RMSE_VAL_, %	1.19	1.17	1.20	1.18	1.18	1.16
**%CY**_**SOLIDS**_						
*Regression coefficients*						
Fat, %	0.80	0.79	0.82	0.80	0.80	0.79
Protein, %	0.96	–	1.03	–	0.98	–
Casein, %	–	1.30	–	1.33	–	1.30
Lactose, %	0.06	0.02	–	–	0.05	0.02
SCS, unit	–	–	– 0.01	0.00	– 0.01	0.00
*Fitting statistics*						
${\text{adjR}}_{\text{CAL}}^{2}$	0.75	0.75	0.75	0.75	0.75	0.75
${\text{R}}_{\text{VAL}}^{2}$	0.75	0.75	0.75	0.75	0.75	0.75
RMSE_VAL_, %	0.47	0.47	0.47	0.47	0.47	0.47
**%CY**_**WATER**_						
*Regression coefficients*						
Fat, %	0.07	0.05	0.19	0.13	0.09	0.06
Protein, %	1.44	–	1.97	–	1.57	–
Casein, %	–	2.02	–	2.59	–	2.14
Lactose, %	0.44	0.37	–	–	0.39	0.33
SCS, unit	–	–	– 0.11	– 0.09	– 0.09	– 0.07
*Fitting statistics*						
${\text{adjR}}_{\text{CAL}}^{2}$	0.32	0.33	0.31	0.33	0.33	0.34
${\text{R}}_{\text{VAL}}^{2}$	0.34	0.35	0.32	0.34	0.34	0.35
RMSE_VAL_, %	1.04	1.03	1.06	1.04	1.04	1.03
**%CY**_**RIPENED**_						
*Regression coefficients*						
Fat, %	0.66	0.64	0.71	0.67	0.68	0.65
Protein, %	1.35	–	1.63	–	1.49	–
Casein, %	–	1.89	–	2.14	–	2.03
Lactose, %	0.19	0.12	–	–	0.13	0.07
SCS, unit	–	–	– 0.10	– 0.09	– 0.10	– 0.08
*Fitting statistics*						
${\text{adjR}}_{\text{CAL}}^{2}$	0.71	0.73	0.72	0.74	0.73	0.74
${\text{R}}_{\text{VAL}}^{2}$	0.70	0.72	0.71	0.73	0.72	0.73
RMSE_VAL_, %	0.61	0.59	0.60	0.58	0.59	0.58

Most of the lactose in milk is lost in the whey in the cheese-making process, and fresh curd usually contains only ~1% of lactose (53), which is bound to the water retained in the curd. Our results reflect this recovery, and show that lactose in combination with fat, protein/casein and SCS mainly affected %CY_CURD_ and %CY_WATER_, whereas the effect on %CY_SOLIDS_ and %CY_RIPENED_ was very small with average regression coefficients of 0.04 and 0.12, respectively (Table 3). Adding lactose to the protein and fat predictors in the %CY_CURD_ model reduced the protein coefficient by about 19%. However, when the overall protein content was replaced in the model by its fractions (caseins and whey proteins) the contribution of lactose to the %CY_CURD_ decreased to 0.09 (Table 4) because the total solids of the whey retained in the curd were also associated with the whey proteins.

**Table 4:** Regression coefficients and fitting statistics (${\text{adjR}}_{\text{CAL}}^{2}$, ${\text{R}}_{\text{VAL}}^{2}$ and RMSE_VAL_) of predicting fresh cheese (%CY_CURD_), ripened cheese (%CY_RIPENED_), cheese solids (%CY_SOLIDS_) and water retained in fresh cheese (%CY_WATER_) of models based on fat, protein fractions and/or non–protein nitrogen (NPN), and/or lactose, and/or somatic cell score (SCS) of processed milk.

	**Models with protein fractions**			**Models with protein fractions, lactose and SCS:**		
	**Fat +caseins**	**Fat** **+caseins** **+whey pr**	**Fat** **+caseins** **+whey pr** **+NPN**	**Fat** **+caseins** **+whey pr** **+lactose**	**Fat** **+caseins** **+whey pr** **+SCS**	**Fat** **+caseins** **+ whey pr** **+NPN** **+lactose** **+SCS**
**%CY**_**CURD**_						
*Regression coefficients*						
Fat, %	0.92	0.85	0.85	0.84	0.87	0.86
α_S1_-CN, %	2.49	4.06	4.25	3.91	3.97	3.79
α_S2_-CN, %	2.39	0.77	1.20	0.83	1.28	1.22
β-CN, %	5.26	5.25	5.47	5.09	5.30	5.11
κ-CN, %	4.79	5.93	6.23	5.90	6.40	6.29
α-LA, %	–	15.38	13.97	14.38	13.87	13.40
β-LG, %	–	– 7.27	– 8.02	– 7.08	– 7.32	– 6.98
NPN, %	–	–	– 0.73	–	–	0.19
Lactose, %	–	–	–	0.09	–	0.07
SCS, unit	–	–	–	–	– 0.07	– 0.07
*Fitting statistics*						
${\text{adjR}}_{\text{CAL}}^{2}$	0.60	0.66	0.66	0.66	0.66	0.66
${\text{R}}_{\text{VAL}}^{2}$	0.61	0.66	0.66	0.66	0.66	0.66
RMSE_VAL_, %	1.20	1.11	1.10	1.11	1.10	1.10
**%CY**_**SOLIDS**_						
*Regression coefficients*						
Fat, %	0.82	0.81	0.81	0.81	0.81	0.82
α_S1_-CN, %	0.89	1.26	1.05	1.28	1.25	1.00
α_S2_-CN, %	1.38	1.14	0.68	1.13	1.15	0.69
β-CN, %	1.37	1.38	1.16	1.41	1.38	1.13
κ-CN, %	2.37	2.63	2.30	2.63	2.63	2.32
α-LA, %	–	2.64	4.17	2.82	2.62	4.17
β-LG, %	–	– 1.70	– 0.90	– 1.74	– 1.70	– 0.78
NPN, %	–	–	0.78	–	–	0.90
Lactose, %	–	–	–	−0.02	–	0.00
SCS, unit	–	–	–	–	0.00	– 0.01
Fitting statistics						
${\text{adjR}}_{\text{CAL}}^{2}$	0.75	0.76	0.76	0.76	0.76	0.76
${\text{R}}_{\text{VAL}}^{2}$	0.75	0.76	0.76	0.76	0.76	0.76
RMSE_VAL_, %	0.47	0.46	0.46	0.46	0.46	0.46
**%CY**_**WATER**_						
*Regression coefficients*						
Fat, %	0.16	0.10	0.09	0.08	0.12	0.09
α_S1_-CN, %	1.14	2.27	2.70	2.03	2.18	2.19
α_S2_-CN, %	0.77	– 0.66	0.29	– 0.54	– 0.12	0.33
β-CN, %	3.94	3.93	4.40	3.66	3.98	3.98
κ-CN, %	3.08	3.87	4.52	3.82	4.33	4.53
α-LA, %	–	12.64	9.55	11.00	11.11	8.65
β-LG, %	–	– 5.29	– 6.94	– 4.98	– 5.36	– 5.68
NPN, %	–	–	– 1.61	–	–	– 0.73
Lactose, %	–	–	–	0.14	–	0.11
SCS, unit	–	–	–	–	– 0.07	– 0.06
Fitting statistics						
${\text{adjR}}_{\text{CAL}}^{2}$	0.32	0.38	0.39	0.39	0.39	0.40
${\text{R}}_{\text{VAL}}^{2}$	0.34	0.40	0.40	0.40	0.40	0.40
RMSE_VAL_, %	1.04	0.99	0.98	0.99	0.98	0.98
**%CY**_**RIPENED**_						
*Regression coefficients*						
Fat, %	0.66	0.64	0.62	0.63	0.66	0.65
α_S1_-CN, %	2.26	2.82	3.28	2.77	2.70	2.99
α_S2_-CN, %	0.81	0.27	1.32	0.29	0.85	1.36
β-CN, %	2.28	2.27	2.78	2.21	2.33	2.62
κ-CN, %	2.28	2.64	3.35	2.63	3.17	3.50
α-LA, %	–	5.07	1.69	4.72	3.41	1.84
β-LG, %	–	– 2.51	– 4.30	– 2.45	– 2.58	– 3.58
NPN, %	–	–	−1.74	–	–	– 0.97
Lactose, %	–	–	–	0.03	–	– 0.01
SCS, unit	–	–	–	–	– 0.08	– 0.07
Fitting statistics						
${\text{adjR}}_{\text{CAL}}^{2}$	0.71	0.74	0.74	0.74	0.75	0.75
${\text{R}}_{\text{VAL}}^{2}$	0.71	0.74	0.74	0.74	0.75	0.75
RMSE_VAL_, %	0.60	0.58	0.57	0.58	0.56	0.56

Moving on to the contribution of milk SCS to %CY traits, high levels of SCC reduced the production efficiency of specific cheeses, such as Parmigiano Reggiano (52) and Cheddar (54) but also in model cheeses produced at individual cow level (55). However, according to Bobbo et al. (23), the effect of SCS on milk production, quality, coagulation, and cheese-making traits is nonlinear because a very low SCS has a slight influence on the cow's performance as the animal could be affected by undetectable intramammary infections. Indeed, Wall et al. (56) suggested that immunocompromised cows might have a very low SCC. Previous studies conducted on commercial productions or where a small number of observations were collected failed to demonstrate this trend.

For this reason, we tested both the linear and quadratic (data not shown) regressions for the effect of SCS on %CY traits, but we observed no differences in the fitting statistics between the two models.

Overall, our results confirm those previously reported by Bobbo et al. (23): a slight negative effect of SCS on %CY_CURD, WATER, RIPENED_, but no effect on %CY_SOLIDS_ in all the models in which SCS was included (Tables 3, 4). Indeed, SCS mainly affects the water retained in the curd and has no effect on %CY_SOLIDS_. In contrast to our observations on the other predictors tested, the SCS coefficient was not lower for %CY_RIPENED_ than for %CY_CURD_, meaning that SCS affects not only fresh cheese yield and recoveries, but also weight loss during ripening.

### Prediction of cheese yield based on fat and the protein fractions

Replacing protein or total casein with the individual milk protein and NPN fractions in the statistical models increased the validation accuracy of the equations obtained (Table 4). The inclusion of protein fractions provided important information on the relative values of each fraction for cheese production, and insights into the mechanisms of the cheese-making process. Protein fractions have been widely studied for their role in the cheese-making process (28, 57), yet to the best of our knowledge this is the first study to quantify the effect of single protein fractions on %CY traits based on a large number of individual model cheese-making procedures.

In comparing the contributions to %CY traits of the four major casein fractions included in the predictive formulas together with fat, we should consider the regression coefficient for total casein (Table 2) as the reference value (3.91 for %CY_CURD_, about a third of which is represented by solids, the remaining two thirds by retained moisture). The regression coefficients of α_S1_-CN and α_S2_-CN were lower than expected (2,49 and 2.39, respectively; Table 4), although the coefficient of α_S2_-CN for %CY_SOLIDS_ was similar to that of total casein (1.38), whereas the coefficient of α_S1_-CN was lower (0.89). Both α_S1_-CN and α_S2_-CN were characterized by %CY_WATER_ coefficients of regression that were much lower than that of total casein (1.14 and 0.77, respectively), a not unexpected result as these caseins have moderate to high hydrophobicity (58).

Conversely, the other casein fractions had much larger regression coefficients for %CY_CURD_ than did total caseins (β-CN = 5.26, κ-CN = 4.79; Table 4). This is due to them having a much larger effect on moisture retention, especially β -CN compared with κ-CN (%CY_WATER_: 3.94 and 3.08, respectively). Given that κ-CN causes loss of stability of casein micelles after rennet-induced proteolysis in para-κ-CN, its high relative weight was expected. The effect on %CY_SOLIDS_ is similar to that of total casein for β -CN (1.37), and much larger (2.37) for κ-CN. This is probably because β -CN increases casein and water retention in the curd (28, 59), whereas κ-CN enhances the recovery of other solids (i.e., fat globules). As further confirmation of its importance in retaining other non-casein solids, the κ-CN coefficient on %CY_SOLIDS_ showed very low variability among the predictive formulas, and a value higher than its own weight, on average 2.48.

Each casein fraction affected moisture retention in fresh cheese yield differently, although these differences tended to decrease during ripening (Table 4) so that when most of the free water had evaporated, the regression coefficients of the major caseins were very similar (2.26–2.28), with the exception of α_S2_-CN, which dropped to well below unity (0.81). Similarly, the regression coefficient of fat (on average %CY_RIPENED_ = 0.64) even dropped to below the average recovery ratio of milk fat in the curd. This shows that not only moisture decreases greatly during ripening, but also that dry mass decreases as a result of the complex biochemical and microbiological processes that characterize cheese ripening (42, 60).

However, when whey proteins were added to the model, the picture changed completely. Although not directly involved in the renneting process, whey proteins contribute to the recovery of nutrients in the curd. Our results agree with those of Bonfatti et al. (18), whose study reported that variation in protein composition affects the cheese-making ability of milk. β-LG had a large, unfavorable effect, and a strong negative coefficient for all the %CY traits, especially %CY_CURD_ and %CY_WATER_, on average – 7.33 and – 5.65, respectively. It is worth noting, however, that this negative effect regarded not only moisture retention, but also the recovery efficiency of milk solids fractions (%CY_SOLIDS_: – 1.70). Previous studies have reported the adverse influence of β-LG on various traits describing the cheese-making process: Cipolat-Gotet et al. found a strong inverse relationship between β-LG and fat and protein recoveries (28), whereas Ketto et al. and Amalfitano et al. found β-LG to be associated with poor coagulation properties (26, 61).

When the overall protein content was replaced by its fractions (caseins and whey proteins) in the model, the contribution of lactose to %CY_CURD_ decreased to 0.09 (Table 4) because the total solids of the whey retained in the curd were also associated with the whey proteins. This may also be related to the positive correlation between lactose and α-LA, the latter being directly involved in lactose synthesis (62).

In contrast, α-LA had a very strong favorable effect on all %CY traits. Bearing in mind that it has a very low concentration and variability in milk (0.09 ± 0.02%), that it is not retained in the curd, and that it is not known to have any direct effect on the cheese-making process, the actual contribution of α-LA to cheese yield is quite small, but it could be considered a marker of favorable/adverse conditions that need to be further investigated. It is worth pointing out that the negative regression coefficients of β-LG and the positive regression coefficients of α-LA remain after the cheese has ripened (%CY_RIPENED_), confirming that the effect of whey proteins is not limited to moisture retention in the fresh curd (Table 4).

The inclusion of whey proteins in the model also caused evident modifications to the regression coefficients of caseins. The only casein fraction remaining unchanged after the inclusion of whey proteins was β-CN. In contrast, the value of the α_s2_-CN coefficient decreased by an average of more than 80% when whey proteins were added as predictors and became negative for %CY_WATER_.

The inclusion of milk NPN compounds in the regression models is also worth some consideration. Although the direct contribution of these substances to curd formation is negligible, adding NPN together with fat and all the protein fractions made a positive contribution to %CY_SOLIDS_ (0.78, and 0.90 after inclusion of UHI; Table 4) and increased the negative effect on %CY_WATER_ (–1.61 and –0.73, respectively), so that the resulting effect on %CY_CURD_ is negative (–0.73) in the first equation, and slightly positive (0.19) after adding UHI. Inclusion of the NPN fraction in the model also affected some of the other regression coefficients, particularly those of α_S2_-CN, which decreased by about 40%.

### Possible practical applications and the need for further research

The regression coefficients obtained with the different models confirm some previous findings, but also contribute new knowledge and shed fresh light on the relationships between milk composition and the mechanisms and efficiency of the cheese-making process. These coefficients may be used for estimating the relative importance of different milk components for the dairy industry.

The ratio of about 3:1 between the regression coefficients of protein and fat (Table 2) represents their relative values in terms of the gross revenue (not profit) of milk destined for cheese-making. In addition, the fat and protein (or casein) coefficients have been widely used in the milk payment system, especially in countries where milk production is mainly destined for cheese manufacture. The destination of milk is also the basis for breeding and selection choices. It is worth noting that ratios equal to or >3:1 are used in the selection indices for most dairy populations, particularly in European countries, but not in the USA (4), where milk is used mainly for direct human consumption.

SCS is the most used udder health indicator worldwide, with very few exceptions, but its weight is highly variable in both payment schemes and selection indices, which reflects different production environments, levels of knowledge, and objectives. SCS is included in selection indices mainly as an indicator of health costs and the durability of the cows (63), whereas in payment schemes it is included as an indicator of low hygiene standards and because it is associated with the efficiency of the cheese-making process. Knowledge and quantification of the latter are still fragmentary and need further research. This information also needs to be incorporated into the selection indices for dairy populations. New insights are now obtainable with the use of differential cell count as an indicator of mastitis (64), but not yet as an indicator of effects on cheese-making.

This study shows that lactose is probably more effective than SCS as an indicator of the relationships between udder health and cheese-making efficiency. However, it is also evident that the complexity of these relationships cannot be captured from the physiological, metabolic, technological, and economic points of view by single indicators, but that both SCS and lactose should be considered together with at least β-LG and NPN, and perhaps also α_S2_-CN, in milk. New UHI should be defined for improving the efficiency of selection to enhance the cow's health and durability, but also the efficiency of the dairy industry, which is the starting point for a more sustainable chain. This last objective could be given a further boost by knowledge of the different impacts and relative importance of protein fractions on cheese-making efficiency. New knowledge in this area is of critical interest to the dairy chain sector.

## Conclusions

In this study, we directly evaluated detailed milk components in relation to their contribution—individually and in combination—to different %CY traits. The large number and variability of individual samples, and direct measurements of %CY traits allowed us to gather information on effectiveness of predictions for application at the dairy cattle population level. Knowledge concerning the relationships between UHI and efficiency of the cheese-making process needs to be integrated with new information on β-LG and milk NPN, and perhaps also α_S2_-CN. The results for the protein fractions provided a much more detailed understanding of the mechanisms that determine cheese yield. Despite the economic importance of the information contained in the detailed protein profile, it is not yet routinely used in milk payment schemes and genetic selection indices as it can only be obtained with expensive, time-consuming methods. This study offers new insights into the quantification of the influence of milk components in composite selection indices with the aim of directly enhancing cheese production.

## Data availability statement

The raw data supporting the conclusions of this article will be made available by the authors, without undue reservation.

## Ethics statement

Ethical review and approval was not required for the study of animals in accordance with the local legislation and institutional requirements. All the dairy cows involved in this study were reared in commercial private farms and were not subjected to any invasive procedures. Milk samples from dairy cows used for the project were collected by technicians of breeders associations during routine milking within current milk-recording schemes (ICAR, International Committee for Animal Recording) and hence certified by local authorities.

## Author contributions

EM wrote the manuscript. CC-G carried out the experimental work and the data analyses and contributed to write the manuscript. GB designed the entire study. AS and GB contributed to conceptualization and funding acquisition. All authors listed contributed to the interpretation of the results. All authors contributed to the article and approved the submitted version.

## Conflict of interest

The authors declare that the research was conducted in the absence of any commercial or financial relationships that could be construed as a potential conflict of interest.

## Publisher's note

All claims expressed in this article are solely those of the authors and do not necessarily represent those of their affiliated organizations, or those of the publisher, the editors and the reviewers. Any product that may be evaluated in this article, or claim that may be made by its manufacturer, is not guaranteed or endorsed by the publisher.
