# Supplementary material for: Prediction of fresh and ripened cheese yield using detailed milk composition and udder health indicators from individual Brown Swiss cows
Source: Front Vet Sci. 2022 Oct 13;9:1012251. doi: 10.3389/fvets.2022.1012251 (PMC9606222; doi:10.3389/fvets.2022.1012251)
Supplement: Supplementary file 1 [file Data_Sheet_1.docx]

Supplementary Material

# Supplementary Tables

**Table S1.** Collinearity diagnostics for two groups of predictor variables (group 1: fat, protein, lactose and SCS; group 2: fat, protein fractions, NPN compounds, lactose and SCS) used for predicting cheese yield traits.

|  | Tolerance | Variance Inflation Factor | Eigen Value | Condition Index |
| --- | --- | --- | --- | --- |
| *Group 1* |  |  |  |  |
| Fat, % | 0.90 | 1.12 | 1.74 | 1.00 |
| Protein, % | 0.87 | 1.15 | 1.09 | 1.26 |
| Lactose, % | 0.72 | 1.39 | 0.70 | 1.58 |
| SCS, unit | 0.72 | 1.39 | 0.47 | 1.92 |
| *Group 2* |  |  |  |  |
| Fat, % | 0.79 | 1.27 | 3.36 | 1.00 |
| α_S1_-CN, % | 0.30 | 3.32 | 1.89 | 1.33 |
| α_S2_-CN, % | 0.47 | 2.14 | 1.09 | 1.76 |
| β-CN, % | 0.38 | 2.60 | 0.97 | 1.86 |
| κ-CN, % | 0.54 | 1.85 | 0.77 | 2.09 |
| α-LA, % | 0.60 | 1.67 | 0.64 | 2.30 |
| β-LG, % | 0.20 | 5.11 | 0.51 | 2.55 |
| NPN, % | 0.19 | 5.33 | 0.40 | 2.90 |
| Lactose, % | 0.47 | 2.12 | 0.31 | 3.32 |
| SCS, unit | 0.69 | 1.45 | 0.07 | 6.73 |

**Table S2.** Pearson product-moment correlations between predictors used in the predictive equations for cheese yield traits.

|  | Protein | Casein | Lactose | SCS | α_S1_-CN | α_S2_-CN | β-CN | κ-CN | α-LA | β-LG | NPN |
| --- | --- | --- | --- | --- | --- | --- | --- | --- | --- | --- | --- |
| Fat | 0.32^***^ | 0.34^***^ | -0.15^***^ | 0.07^*^ | 0.37^***^ | 0.16^***^ | 0.24^***^ | 0.25^***^ | 0.10^***^ | 0.27^***^ | 0.04 |
| Protein |  | 0.99^***^ | -0.17^***^ | 0.22^***^ | 0.85^***^ | 0.69^***^ | 0.81^***^ | 0.67^***^ | 0.20^***^ | 0.63^***^ | 0.57^***^ |
| Casein |  |  | -0.09^**^ | 0.17^***^ | 0.86^***^ | 0.69^***^ | 0.82^***^ | 0.66^***^ | 0.24^***^ | 0.64^***^ | 0.49^***^ |
| Lactose |  |  |  | -0.51^***^ | -0.06^*^ | -0.16^***^ | 0.01 | -0.18^***^ | 0.15^***^ | -0.14^***^ | -0.36^***^ |
| SCS |  |  |  |  | 0.11^***^ | 0.16^***^ | 0.09^**^ | 0.24^***^ | -0.13^***^ | 0.08^**^ | 0.35^***^ |
| α_S1_-CN |  |  |  |  |  | 0.60^***^ | 0.60^***^ | 0.61^***^ | 0.25^***^ | 0.66^***^ | 0.37^***^ |
| α_S2_-CN |  |  |  |  |  |  | 0.49^***^ | 0.46^***^ | 0.44^***^ | 0.49^***^ | 0.35^***^ |
| β-CN |  |  |  |  |  |  |  | 0.38^***^ | 0.19^***^ | 0.47^***^ | 0.47^***^ |
| κ-CN |  |  |  |  |  |  |  |  | 0.17^***^ | 0.50^***^ | 0.35^***^ |
| α-LA |  |  |  |  |  |  |  |  |  | 0.27^***^ | -0.23^***^ |
| β-LG |  |  |  |  |  |  |  |  |  |  | -0.13^***^ |

**P* < 0.05; ***P* < 0.01; ****P* < 0.001.
